# Atlas-based white matter analysis in individuals with velo-cardio-facial syndrome (22q11.2 deletion syndrome) and unaffected siblings

**DOI:** 10.1186/1744-9081-8-38

**Published:** 2012-08-01

**Authors:** Petya D Radoeva, Ioana L Coman, Kevin M Antshel, Wanda Fremont, Christopher S McCarthy, Ashwini Kotkar, Dongliang Wang, Robert J Shprintzen, Wendy R Kates

**Affiliations:** 1 Department of Neuroscience and Physiology, SUNY Upstate Medical University, Syracuse, NY, USA; 2 Department of Psychiatry and Behavioral Sciences, SUNY Upstate Medical University, Syracuse, NY, USA; 3 Department of Public Health and Preventive Medicine, SUNY Upstate Medical University, Syracuse, NY, USA; 4 The Virtual Center for Velo-Cardio-Facial Syndrome, http://www.vcfscenter.com, Manlius, NY, USA; 5 Program in Neuroscience, SUNY Upstate Medical University, Syracuse, NY, USA; 6 Department of Psychiatry and Behavioral Sciences, SUNY Upstate Medical University, 750 East Adams Street, Syracuse, NY, 13210, USA

**Keywords:** VCFS, 22q11.2 deletion, DTI, White matter, LDDMM

## Abstract

**Background:**

Velo-cardio-facial syndrome (VCFS, MIM#192430, 22q11.2 Deletion Syndrome) is a genetic disorder caused by a deletion of about 40 genes at the q11.2 band of one copy of chromosome 22. Individuals with VCFS present with deficits in cognition and social functioning, high risk of psychiatric disorders, volumetric reductions in gray and white matter (WM) and some alterations of the WM microstructure. The goal of the current study was to characterize the WM microstructural differences in individuals with VCFS and unaffected siblings, and the correlation of WM microstructure with neuropsychological performance. We hypothesized that individuals with VCFS would have decreased indices of WM microstructure (fractional anisotropy (FA), axial diffusivity (AD) and radial diffusivity (RD)), particularly in WM tracts to the frontal lobe, and that these measures would be correlated with cognitive functioning.

**Methods:**

Thirty-three individuals with VCFS (21 female) and 16 unaffected siblings (8 female) participated in DTI scanning and neuropsychological testing. We performed an atlas-based analysis, extracted FA, AD, and RD measures for 54 WM tracts (27 in each hemisphere) for each participant, and used MANOVAs to compare individuals with VCFS to siblings. For WM tracts that were statistically significantly different between VCFS and siblings (*p*_*FDR*_ < 0.05), we assessed the correlations between DTI and neuropsychological measures.

**Results:**

In VCFS individuals as compared to unaffected siblings, we found decreased FA in the uncinate fasciculus, and decreased AD in multiple WM tracts (bilateral superior and posterior corona radiata, dorsal cingulum, inferior fronto-occipital fasciculus, superior longitudinal fasciculus, superior cerebellar peduncle, posterior thalamic radiation, and left anterior corona radiata, retrolenticular part of the internal capsule, external capsule, sagittal stratum). We also found significant correlations of AD with measures of executive function, IQ, working memory, and/or social cognition.

**Conclusions:**

Our results suggest that individuals with VCFS display abnormal WM connectivity in a widespread cerebro-anatomical network, involving tracts from/to all cerebral lobes and the cerebellum. Future studies could focus on the WM developmental trajectory in VCFS, the association of WM alterations with psychiatric disorders, and the effects of candidate 22q11.2 genes on WM anomalies.

## Background

Velo-cardio-facial syndrome (VCFS; MIM#192430) is a genetic disorder caused by a microdeletion of a portion of the 11.2 band (spanning approximately 40 genes in most cases) of one copy of chromosome 22. The phenotypic spectrum of VCFS includes cardiac malformations, palatal anomalies with speech impairment, endocrine and immune problems [1]. Notably, individuals with VCFS often have cognitive deficits in attention, working memory, executive function, visuospatial perception, math abilities, and reading comprehension [2,3]. In addition to cognitive deficits, individuals with VCFS present with emotion dysregulation [2,4,5], modestly difficult temperament [6], and social withdrawal [7,8]. High prevalence of psychiatric disorders [9] has been reported in VCFS across multiple studies, including autism spectrum disorder (ASD) [10,11], attention deficit hyperactivity disorder (ADHD) [9], schizophrenia/schizoaffective disorder [12,13], anxiety disorders [14], and mood disorders [9].

Neuroimaging studies of individuals with VCFS have found volumetric reductions, including reduction in subregions of the frontal lobe, decreased volumes of the gray and white matter in the parietal, temporal, and occipital lobes, smaller hippocampus (bilaterally), and smaller cerebellum (for meta-analysis see [15]). In addition to volumetric reductions, specific structural abnormalities have been described in both the gray and white matter of individuals with VCFS, including white matter hyperintensities, cavum septum pellucidum/vergae, pachygyria, polymicrogyria, cortical dysgenesis or dysplasia, and Arnold-Chiari malformation [16-19]; for review, see [1].

Diffusion tensor imaging (DTI) has also been used to evaluate the microstructure of WM in VCFS. Several measures can be derived from DTI scans, including fractional anisotropy (FA), axial diffusivity (AD) and radial diffusivity (RD). In general, decreases in FA are associated with various WM neuropathologies, including demyelination, ischemia, and inflammation. While FA is a sensitive measure of WM microstructural changes, it is not very specific as to the type/cause of WM alteration [20]. Additional DTI measures, including axial diffusivity and radial diffusivity, can better characterize the specific types of WM microstructural changes, and it has been argued that such measures should be routinely included in DTI studies [20]. Increases in RD, for example, have been associated with demyelination [21], while decreases in AD have been correlated with increased axonal damage [21,22].

With the exception of one report [23], all previously published DTI studies of individuals with VCFS have focused exclusively on FA. Alterations in FA have been reported in VCFS-affected individuals in frontal, temporal and parietal areas, including anomalous tracts between frontal-temporal and frontal-parietal lobes [24,25], and the posterior limb of the internal capsule [26]. Associations between alterations in FA and neuropsychological/psychiatric function in VCFS have also been reported for schizotypy [26], arithmetic abilities [24] and (along with AD alterations) spatial attention [23].

While these studies are important initial steps in the study of white matter microstructure in VCFS and brain-cognition correlations, several were limited, to some extent, by small sample sizes [26], wide age ranges [25] and a primary focus on FA [24-26]. As noted above, analyses of associations between DTI measures and neuropsychological data were also limited, in that many cognitive functions that are impaired in VCFS (e.g., memory, executive functioning, social cognition) have not yet been examined in relationship to white matter microstructure in this disorder. A more detailed study, therefore, of additional measures in multiple white matter tracts, in association with a wider range of cognitive functions, could better elucidate the underlying neuropathology of white matter changes in VCFS.

In our current study, therefore, we utilized a novel DTI analysis method— atlas-based whole brain white matter analysis [27], to assess the microstructure (including FA, AD and RD measures) of a large number of white matter tracts in 33 individuals with VCFS and their unaffected siblings. Our goals were (1) to increase the power to detect microstructural WM alterations in VCFS by using a larger sample size of individuals with VCFS; (2) to investigate the relative contributions of AD and RD to WM alterations in VCFS; (3) to evaluate the correlations of WM microstructure with a wide variety of neuropsychological standardized tests, including attention, working memory, executive functioning, social cognition, and psychiatric measures. Based on the previous VCFS literature, we hypothesized that relative to their siblings, individuals with VCFS would display alterations in FA, RD and AD which would be distributed in frontal, parietal and temporal areas and the internal capsule. We further hypothesized that psychiatric measures would correlate with DTI measures in the internal capsule. Studies of WM microstructural underpinnings of cognitive function in the non-VCFS population led us to further hypothesize the following associations: executive function with cortico-subcortical tracts [28], superior longitudinal fasciculus (SLF), and superior corona radiata (SCR) [29]; working memory with SLF [30], SCR, and posterior corona radiata (PCR) [29]; and social cognition/socialization with uncinate fasciculus (UNC) [31], SLF, posterior limb of the internal capsule (PLIC), anterior limb of the internal capsule (ALIC) and anterior thalamic radiation (ATR) [32].

## Materials and methods

### Participants

In this paper, we are reporting on the data collected on 49 individuals, who are participants in a longitudinal study of VCFS [33,34]. The study was approved by the IRB at SUNY Upstate Medical University, and informed consent was obtained from the participants and/or their parents. We included data from all individuals who participated in the study between December, 2008 and February, 2011 on whom we collected DTI as well as neuropsychological data. DTI data from four additional individuals with VCFS were excluded due to poor image quality or severe motion/scanning artifacts (see Section *DTI processing and data analysis*). The VCFS diagnosis was confirmed with fluorescence *in situ* hybridization (FISH). This sample includes 33 individuals with VCFS (12 male), and 16 unaffected siblings (8 male)^a^, with average age for the VCFS group 17.7 (SD = 1.8) and for the sibling group 18.0 (SD = 1.7) (Table 1 and Table 2). Although all of the unaffected siblings who participated in the larger longitudinal study had a matching brother or sister with VCFS, four of the siblings reported here did not, because imaging data from his/her counterpart with VCFS could not be acquired/used due to braces (n = 1), claustrophobia (n = 1), severe scoliosis (n = 1) or severe motion/scanning artifacts (n = 1). All of the participants were Caucasian except one participant with VCFS and one sibling who were Asian. The average full-scale IQ was 73 (SD = 12.9, ranging between 44 and 98) for the individuals with VCFS and 113 (SD = 11.5, ranging between 98 and 141) for the siblings. 

**Table 1:** Demographics of the participants

	**VCFS**	**Siblings**	**P-value**
	**(N = 33)**	**(N = 16)**	
**Gender (N, % female)**	21 (64%)	8 (50%)	N.S.
**Race (Caucasian/Asian)**	32/1	15/1	N.S.
**Age, in years (+/− SD)**	17.7 (1.8)	18.0 (1.7)	N.S.
**FSIQ (+/− SD)**	73 (12.9)	113 (11.5)	< 0.001

**Table 2:** **Number (and percent) of participants with psychiatric diagnoses in the current study based on the Schedule for Affective Disorders and Schizophrenia for School-Age Children—Present and Lifetime Version (K-SADS-PL)**[35]

**Psychiatric Diagnoses**	**VCFS (N = 33)**	**Siblings (N = 16)**
	**N (%)**	**N (%)**
Schizophrenia	1 (3.0)	0 (0)
Major depressive disorder (includes NOS)	6 (18.2)	0 (0)
Bipolar disorder	1 (3.0)	0 (0)
Anxiety disorder (includes generalized, overanxious, separation and panic)	7 (21.2)	0 (0)
Simple or social phobia	11 (33.3)	3 (18.75)
ADHD	10 (30.3)	1 (6.25)
Enuresis	1 (3.0)	0 (0)
Chronic motor or vocal tic disorder	2 (6.1)	0 (0)
Oppositional defiant disorder	3 (9.1)	0 (0)
Any disorder listed above	20 (60.6)	3 (18.75)

### DTI acquisition

The DTI scans were acquired on a 1.5 T Philips Interra scanner (release 11) equipped with a Sense Head coil to improve the signal strength and the signal-to-noise ratio. A multi-slice, single-shot EPI (SENSE factor = 2.0), spin echo sequence (TR/TE = 8197/76 ms) was used to obtain 70 axial slices with no slice gap and 2.5 mm nominal isotropic resolution (FOV = 240 × 240, data matrix = 96 × 96, zero-filled and reconstructed to 256 × 256). Diffusion weighting was applied along 15 directions [36] with a *b* factor = 800 s/mm^2^. One minimally weighted volume (b0) was acquired within each DTI dataset. The total scan time to acquire one DTI dataset (15 DW and 1 b0 images) was 2 min 11 s. The total time, including image reconstruction, to acquire 4 DTI datasets in a scan session (for each participant) was approximately 9 minutes.

### DTI processing and data analysis

The data were downloaded from the scanner, transferred and processed using DTIStudio 3.0.2, DiffeoMap 1.7.1, and ROI Editor 1.4.2 (https://www.mristudio.org/, [37]) on a 64-bit Dell PC, running Windows 7 operating system. First, by utilizing a mutual information algorithm [38], all diffusion weighted images from a study (the four repeats) were coregistered to the same reference volume, the b0 volume of the first repeat. Axial slices with severe scanning and motion artifacts were excluded via automatic outlier slice rejection in DTIStudio (with relative error > 3%), and through visual inspection. The diffusion weighted images (for each diffusion direction) were then averaged, and the average set was used for further analysis.

**Figure 1:**
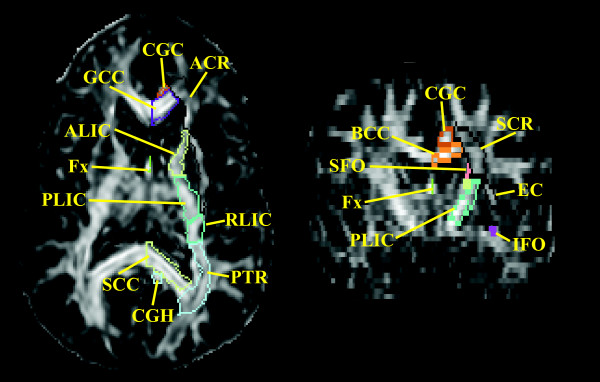
**White matter tracts analyzed in the current report represented on the FA map of one individual with VCFS. ***Abbreviations*: ***ACR***: Anterior corona radiata; ***ALIC***: Anterior limb of the internal capsule; ***BCC***: Body of the corpus callosum; ***CGC***: Cingulum (cingulate gyrus); ***CGH***: Cingulum (hippocampus); ***EC***: External capsule; ***Fx***: Fornix (column and body of the fornix); ***GCC***: Genu of the corpus callosum; ***IFO***: Inferior fronto-occipital fasciculus; ***PLIC***: Posterior limb of the internal capsule; ***PTR***: Posterior thalamic radiation; ***SCR***: Superior corona radiata; ***SS***: Sagittal stratum; ***RLIC***: Retrolenticular part of the internal capsule. For the full list of WM tracts analyzed in the current study, see the List of Abbreviations.

Tensor estimation was then performed, and Fractional Anisotropy (FA), Axial Diffusivity (AD), Radial Diffusivity (RD), and b0 maps were computed and saved (while applying a skull-stripped mask generated in ROI Editor for the b0 image of each participant). The FA and b0 maps of each participant were then used for Large Deformation Diffeomorphic Metric Mapping (LDDMM) [27], and regions of interest (ROIs) were generated for each participant as follows. The b0 and FA maps of each participant were first transformed linearly (using affine Automated Image Registration (AIR) transformation, with trilinear interpolation) and then non-linearly (using LDDMM, with cascading alpha of 0.01, 0.005, and 0.002), in order to match as well as possible the corresponding Johns Hopkins University MNI-space single participant skull-stripped templates (JHU_MNI_SS_b0_ss and JHU_MNI_SS_FA_ss). A detailed atlas of the white matter tracts and gray matter ROIs had been previously constructed by [27] based on the data from the participant used in the Johns Hopkins University MNI-space single participant skull-stripped templates. Next, the inverse transformation algorithms (inverse LDDMM and then inverse AIR) were applied to the ROI atlas (JHU_MNI_SS_WMPM_TypeII), in order to obtain ROIs that are within each participant's original brain space. To ensure the proper execution of the algorithms, the ROIs generated were visually inspected for accuracy. The mean FA, AD, and RD values for the ROIs were then extracted in ROI Editor. For further analyses, we focused on the measures FA, AD, and RD of all available white matter tract ROIs (27 tracts in each hemisphere; for a complete list see the list of Abbreviations at the end of the paper). Sample ROIs are shown in Figure 1. 

### Neuropsychological Testing

As part of the larger longitudinal study, the participants were tested with a wide array of neuropsychological tests. The Wechsler Intelligence Scale for Children— Third Edition (*WISC-III*) [39] was administered to participants under 17 years of age, and the Wechsler Adult Intelligence Scale (*WAIS-III)*[40] to participants 17 years of age or older.

### Attention, Memory and Executive Functioning Measures

*Digit Span* was evaluated as part of WISC-III or WAIS-III. Forward and Backward z-scores were used for further analyses.

*Visual Span Test*[41]: In this computerized instrument, each participant was asked to reproduce an increasing number of patterns of squares displayed on a computer screen [10]. This test evaluates spatial/non-verbal working memory, and the Forward and Backward Visual Span Z-Scores were used.

*CVLT (California Verbal Learning Test)*[42]: All participants completed the CVLT, which evaluated verbal learning and memory. The CVLT (1) T-scores for List A, trials 1–5; and (2) standard score for List A, trial 5 [33] were used for further analyses.

*Wisconsin Card Sorting Test (WCST)*[43]: Each participant completed the WCST as part of evaluation of executive functioning and, more specifically, cognitive flexibility. The Perseverative Error Standard Score and the Non-Perseverative Error Standard Score of WCST were used for further analyses.

*BRIEF (Behavior Rating Inventory of Executive Functioning)*[44]: Parents completed the BRIEF or BRIEF-A questionnaires. The T-scores of the (1) Metacognition Index (assessing initiation, organization, planning, monitoring, and working memory); and (2) the Behavioral Regulation Index (evaluating inhibition, shift, and cognitive control); were included for further analyses.

### *Social Cognition/Skills Measures*

The following instruments were used:

*Emotional Recognition Test*[45]: In this computerized test, each participant was asked to discriminate between happy, sad and neutral faces. The total number of correct responses was used for further analysis.

*BASC-2* (Behavior Assessment System for Children, Second Edition): Parents completed the BASC-2 [46], which contains 150 items that are rated on a 4-point scale. Scores were then derived for a variety of domains such as social skills, withdrawal, conduct problems. The T-scores of the BASC-2 social skills domain, atypicality, and anxiety were used for analysis in this report.

*Vineland-II* (Vineland Adaptive Behavior Scales, Second Edition): Parents were interviewed with Vineland-II [47], evaluating various aspects of the child's behavior, including social skills (socialization subdomain).

*SRS (Social Responsiveness Scale)*: Parents completed the SRS, which consists of 65 items [48,49], and measures aspects of social awareness, social cognition, social communication, social motivation, and autistic mannerisms, and provides a total score. The items are slightly different (but comparable) for children aged 18 or younger vs. adults (19 or older). Since norms, T-scores, and domain classification are provided only for the child/adolescent scale (but not for the adult version), the total raw score was used for further analysis for all participants.

*CGAS (Children's Global Assessment Scale)*[50]: A clinician evaluated the global functioning of each participant (based on an interview with the parent and the child), and completed the CGAS.

### Statistical Analysis

The Shapiro – Wilk Test of Normality was used to investigate the distribution of all data (see results, below). Three MANOVAs were conducted with dependent variables mean FA (or AD or RD) values (in each of the tracts) and independent variable Group (VCFS vs. siblings), using SPSS 18 (http://www.spss.com/). FDR (false discovery rate) correction for multiple comparisons was applied in the program R (http://www.r-project.org/) on the p-values from each of the three MANOVAs [51]. For tracts that showed significant differences between the participants with VCFS and controls (*p*_FDR_ < 0.05), Pearson's correlations were performed (in SPSS) between the DTI measures of the tracts, and each of the neuropsychological measures (described above), across all of the study participants. Since multiple correlations were performed, the p-values of the correlations were also FDR-corrected.

As noted in the background, individuals with VCFS have a higher prevalence of certain brain abnormalities, including *cavum septum pellucidum/vergae*. Four VCFS participants in our current sample have this variant as evaluated by a neuroradiologist. The presence of *cavum septum pellucidum/vergae* seems to be associated with an alteration of the anatomy of the fornix, such that the columns and body of the fornix do not join in the midline and seem to run separately within the left and right hemispheres between the cavum septum pellucidum and the lateral ventricles [52]. Thus, the automated fornix measures in the current study might not be valid for individuals with *cavum septum pellucidum/vergae*, so we excluded these four individuals from the analyses *only* of the fornix measures.

## Results

**Figure 2:**
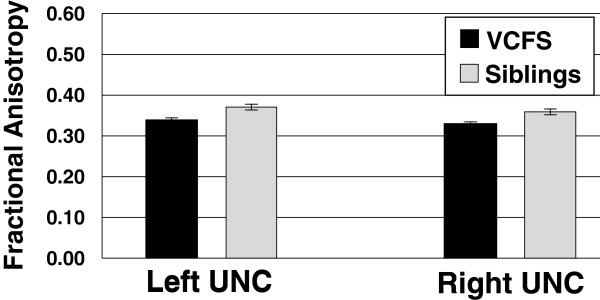
**Significant Differences in Fractional Anisotropy ( *p***_***FDR***_ **< 0.05) in individuals with VCFS vs. siblings in the left and right uncinate fasciculi (UNC, L and UNC, R respectively).** Error bars show SE.

**Figure 3:**
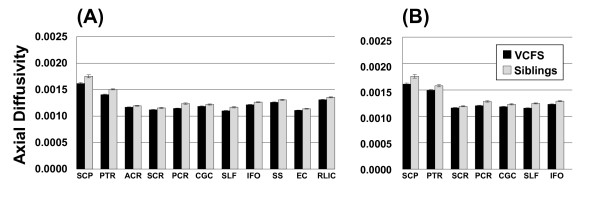
**Significant Differences in Axial Diffusivity ( *p***_***FDR***_ **< 0.05) in individuals with VCFS (black) vs. siblings (grey) in the (A) left; or (B) right sides of the brain.** Error bars show SE. *Abbreviations*: ***SCP***: Superior cerebellar peduncle; ***PTR***: Posterior thalamic radiation; ***ACR***: Anterior corona radiata; ***SCR***: Superior corona radiata; ***PCR***: Posterior corona radiata; ***CGC***: Cingulum (cingulate gyrus); ***SLF***: Superior longitudinal fasciculus; ***IFO***: Inferior fronto-occipital fasciculus; ***SS***: Sagittal stratum; ***EC***: External capsule; ***RLIC***: Retrolenticular part of the internal capsule.

The MANOVAs demonstrated that the FA in the left and right uncinate fasciculi (see Figure 2), and RD in the right posterior corona radiata (PCR) differed between participants with VCFS and siblings (*p*_*FDR*_ < 0.05). Furthermore, a widely distributed network of tracts showed significantly lower AD in individuals with VCFS as compared to siblings (*p*_*FDR*_ < 0.05) (see Figure 3), including tracts terminating in the *parietal/occipital* (posterior thalamic radiation, PTR; posterior corona radiata, PCR; retrolenticular part of the internal capsule, RLIC; sagittal stratum, SS), and/or *frontal* cortices (superior corona radiata, SCR; anterior corona radiata, ACR); as well as *fronto-parietal/occipital* (inferior fronto-occipital fasciculus, IFO; superior longitudinal fasciculus, SLF; external capsule, EC); *fronto-temporal* (cingulum, CGC); and *cerebellar* connections (superior cerebelar peduncle, SCP). For reasons described below, both the uncorrected and FDR-corrected P-values from the MANOVAs are included in Appendix 1. The majority of the WM measures had normal distributions in both the VCFS and the control groups. However, some of the distributions were not normal, and there were outliers for some of the tracts. Therefore, as a follow-up, we conducted non-parametric analysis (Mann–Whitney U tests), which is less sensitive to outliers and can appropriately be used for non-normally distributed data, and compared the DTI measures of the tracts (for VCFS vs. controls), and corrected the p-values using FDR. All the tracts summarized in Figures 2 and 3 remained significant with this non-parametric analysis. RD in the right posterior corona radiata (PCR) was not significant in this analysis, and was dropped from further analyses. In addition, several tracts that had non-normal distributions showed significant differences between patients and controls: namely, the AD of the left and right ML, left UNC, right MCP, and right RLIC (data not shown).

AD values were significantly correlated with several neuropsychological and psychiatric measures across all participants (Tables 3 and 4). AD values in *fronto-parietal/occipital circuits* (SLF, IFO) correlated with measures of *working memory*, *executive functioning*, and *social cognition*. In addition measures of *executive functioning* correlated with AD in PCR and PTR bilaterally. Overall measures of cognitive skills (intelligence, VIQ and PIQ) correlated with the majority of the studied tracts, which may be expected, since IQ is a composite assessment of multiple domains including attention, working memory, verbal comprehension, and processing speed (Tables 3 and 4).

**Table 3:** Correlations between neuropsychological measures and the Axial Diffusivity of white matter tracts in the Left Hemisphere across all study participants

**Domain**	**Measure**	**Left Hemisphere**									
		**Frontal/Temp**			**Parietal/Occipital A/P Long Tracts Cer**						
		**CGC**	**SCR**	**RLIC**	**EC**	**PTR**	**PCR**	**SLF**	**IFO**	**SS**	**SCP**
**Memory & Attention**	Digit Span FW	0.19	0.15	0.13	0.29	0.19	0.07	0.14	0.22	0.19	0.35^*^
	Digit Span BW	0.15	0.15	0.17	0.08	0.37^*^	0.20	0.23	0.20	0.21	0.34
	Visual Span FW	0.33	0.28	0.31	0.25	0.36^*^	0.47^**^	0.40^*^	0.28	0.29	0.25
	Visual Span BW	0.34	0.28	0.43^*^	0.29	0.52^**^	0.48^**^	0.47^**^	0.40^*^	0.38^*^	0.27
	CVLT_List A, Trials 1–5	0.07	0.18	0.08	0.09	0.30	0.35	0.37^*^	0.14	0.16	0.21
**Executive Function**	WCST Perseverative Error	0.18	0.30	0.33	0.21	0.45^**^	0.43^*^	0.50^**^	0.27	0.33	0.34
	BRIEF Behavioral Regulation	0.00	−0.18	−0.17	−0.24	−0.42^*^	−0.28	−0.30	−0.23	−0.16	−0.30
	BRIEF Metacognition	−0.15	−0.15	−0.22	−0.26	−0.51^**^	−0.31	−0.34	−0.32	−0.18	−0.27
**Social Cognition**	BASC Social Skills	0.08	0.16	0.11	0.23	0.24	0.28	0.29	0.17	0.21	0.26
**Socialization**	Vineland Soc Skills	0.01	0.19	0.21	0.28	0.45^**^	0.37^*^	0.40^*^	0.27	0.33	0.29
	SRS	−0.08	−0.25	−0.26	−0.27	−0.49^**^	−0.42^*^	−0.42^*^	−0.30	−0.25	−0.33
**Emotion**	Emotion Recognition	0.17	0.24	0.33	0.20	0.30	0.27	0.33	0.20	0.10	0.22
**Psychiatric**	BASC Anxiety	−0.06	−0.21	−0.08	−0.07	−0.31	−0.28	−0.27	−0.11	−0.19	−0.34
**Symptoms**	BASC Atypicality	−0.01	−0.30	−0.25	−0.24	−0.37^*^	−0.35	−0.36^*^	−0.27	−0.15	−0.30
	CGAS	0.07	0.24	0.06	0.12	0.39^*^	0.33	0.37^*^	0.21	0.16	0.26
**Overall**	Verbal IQ	0.32	0.34	0.47^**^	0.35^*^	0.52^**^	0.54^**^	0.55^**^	0.36^*^	0.43^*^	0.43^*^
**Cognition**	Performance IQ	0.39^*^	0.38^*^	0.50^**^	0.35^*^	0.62^***^	0.55^**^	0.54^**^	0.38^*^	0.45^*^	0.42^*^

The subset of WM tracts included in this table had significantly different AD between individuals with VCFS and unaffected siblings, *p*_*FDR*_<0.05. The R values for the correlations are given, and corresponding FDR-corrected P-values are indicated with a superscript according to the legend below the table. No significance was found for the Non-Perseverative Error Standard Score of WCST, the standard score for List A, trial 5, and AD of the ACR tract in the left hemisphere: thus, these measures were not included in this table.

***AbbreviationsBW***: Backward; ***FW***: Forward; ***CVLT***: California Verbal Learning Test; ***WCST***: Wisconsin Card Sorting Test; ***BRIEF***: Behavior Rating Inventory of Executive Functioning; ***SRS***: Social Responsiveness Scale; ***BASC***: Behavior Assessment System for Children; ***CGAS***: Children's Global Assessment Scale; ***IQ:*** Intelligence Quotient. Abbreviations for the white matter tracts are given in the list of Abbreviations at the end of the paper. ***A/P Long Tracts***: Anterior-Poster Long Tracts; ***Cer***: cerebellum.

* *p*_*FDR*_ < 0.05.

^**^p_*FDR*_ < 0.01.

^***^p_*FDR*_ < 0.001.

**Table 4:** Correlations between neuropsychological measures and Axial Diffusivity of white matter tracts in the Right Hemisphere across all study participants

**Domain**	**Measure**	**Right Hemisphere**						
		**Frontal/Temp Parietal/Occipital A/P Long Tracts Cer**						
		**CGC**	**SCR**	**PTR**	**PCR**	**SLF**	**IFO**	**SCP**
**Memory & Attention**	Digit Span FW	0.23	0.20	0.25	0.17	0.16	0.25	0.36^*^
	Digit Span BW	0.24	0.12	0.38^*^	0.26	0.30	0.36^*^	0.44^*^
	Visual Span FW	0.42^*^	0.38^*^	0.29	0.48^**^	0.47^**^	0.48^**^	0.25
	Visual Span BW	0.38^*^	0.24	0.25	0.51	0.57^**^	0.61^***^	0.29
	CVLT_List A, , Trials 1–5	0.18	0.17	0.29	0.36^*^	0.38^*^	0.25	0.26
**Executive Function**	WCST Perseverative Error	0.29	0.29	0.37^*^	0.48^**^	0.58^**^	0.48^**^	0.32
	BRIEF Behavioral Regulation	−0.17	−0.31	−0.28	−0.40^*^	−0.48^**^	−0.34	−0.17
	BRIEF Metacognition	−0.22	−0.36^*^	−0.30	−0.45^**^	−0.53^**^	−0.41^*^	−0.18
**Social Cognition**	BASC Social Skills	0.14	0.14	0.23	0.29	0.43^*^	0.24	0.22
**Socialization**	Vineland Soc Skills	0.17	0.22	0.27	0.38^*^	0.54^**^	0.41^*^	0.31
	SRS	−0.24	−0.34	−0.27	−0.49^**^	0.41^*^	−0.44^*^	−0.27
**Emotion**	Emotion Recognition	0.24	0.15	0.17	**0.28**	0.38^*^	0.41^*^	0.23
**Psychiatric**	BASC Anxiety	−0.13	−0.23	−0.19	−0.34	−0.39^*^	−0.22	−0.31
**Symptoms**	BASC Atypicality	−0.16	−0.38^*^	−0.26	−0.44^*^	−0.48^**^	−0.35^*^	−0.23
	CGAS	0.11	0.29	0.31	0.44^*^	0.57^**^	0.39^*^	0.33
**Overall**	Verbal IQ	0.44^*^	0.24	0.46^**^	0.48^**^	0.60^***^	0.52^**^	0.47^**^
**Cognition**	Performance IQ	0.49^**^	0.37^*^	0.45^**^	0.58^**^	0.63^***^	0.62^***^	0.47^**^

^*^p_*FDR*_ < 0.05.

^**^p_*FDR*_ < 0.01.

^***^p_*FDR*_ < 0.001.

The subset of WM tracts included in this table had significantly different AD between individuals with VCFS and unaffected siblings (*p*_*FDR*_ < 0.05). For abbreviations and further details, see the legend of Table 3.

## Discussion

### Differences between individuals with VCFS and siblings

Our current findings are partially consistent with some of the data reported previously, and further suggest that a more widely distributed set of tracts, including cortico-cortical and cortico-subcortical tracts, may show abnormalities in VCFS than previously reported. Differences in individuals with VCFS and controls have been reported previously, for FA putatively in the SLF and ILF [23,25], pre- and post-central gyri (likely reflecting cortico-spinal tract alterations) [25], radial diffusivity likely in SLF and the fasciculus occipito-frontalis and axial diffusivity possibly in SCR, PCR, and RLIC/PLIC (according to Figure 3 in [23]). Our findings of group differences in a greater number of tracts than previous studies may be related to increased statistical power due to larger sample size and novel data analysis method. Our study had 33 participants with VCFS while the previous DTI studies have included between 11 and 19 individuals with VCFS [23-26]. Furthermore, the atlas-based analysis (ABA) that we utilized has higher statistical power than VBM (which has been used in previous DTI studies of VCFS), because fewer comparisons are conducted in ABA than in VBM (i.e., 27 tracts per hemisphere vs. thousands of voxels across the brain), and ABA does not need to utilize spatial, isotropic blurring, which is often applied in VBM and can potentially obscure differences and introduce noise within WM tracts of close spatial proximity [27]. Furthermore, some of the differences in our results relative to previous DTI findings in VCFS may be related to age effects. For example, the VCFS sample of [23] included a younger age group-- children aged 7 to 14. Here, we found fewer alterations in RD than Simon and colleagues (2008) [23], and it is possible that changes in myelination as the brain matures may account for some of these effects.

Interestingly, our findings of lower AD in multiple WM tracts (in contrast to RD differences) in VCFS relative to controls may suggest axonal damage/loss, or axonal fiber maldevelopment in VCFS, rather than demyelination [22] as a neuropathological correlate of the WM alterations in VCFS individuals. Several 22q11.2 genes (COMT, PRODH, ZDHHC8, DGCR6) are involved in at least 3 major neurotransmitter systems (dopaminergic, glutamatergic and GABAergic) [53-56]. Thus, it is likely that haploinsufficiency, SNPs on the remaining copy of these genes, and/or gene-gene interactions can result in changes of synaptic functioning, axonal maldevelopment or loss, and could ultimately underlie the AD decreases observed in the current study. Several myelin-related genes, including PIK4CA, SNAP29, and RTN4R [57-60] are also located in the 22q11.2 region and, therefore, could affect myelination, and possibly the RD and FA measures. Accordingly, haploinsufficiency of one or more of those genes could account for our current findings. Future genetics studies (e.g., focused on SNPs on the remaining copy of 22q11.2 genes in VCFS individuals) would be crucial in elucidating the roles of specific genes in the WM microstructural deficits observed in VCFS.

Furthermore, we found that there was a somewhat larger number of WM tracts with significantly lower AD in individuals with VCFS (as compared to controls) in the left hemisphere (11 tracts) vs. the right hemisphere (7 tracts) (see Figure 3), particularly in tracts to the frontal, and parietal lobes (ACR, SS, EC, RLIC). These results may be relevant to the findings of reduced laterality preference in VCFS [61], although we cannot directly address this relationship in our current study since we do not have detailed laterality preference/handedness measures on the VCFS and control participants.

### DTI correlates of neuropsychological performance

Several studies have reported *working memory* and *executive functioning* deficits in VCFS [62-64]. Neuroanatomical correlates of working memory in typically developing children (as rated by their parents) include frontal gray matter (GM) volume [65], as well as neural activation in frontal and/or parietal areas in VCFS (as evaluated by fMRI) [66,67]. Our current results demonstrate that working memory (and executive functioning) is associated with WM microstructure in PTR and PCR (i.e., abnormal connections to the parietal/occipital cortex), as well as SLF and IFO (abnormal frontal-occipital/parietal connectivity).

A wide variety of brain regions have been shown to subserve *social cognition* (for review, see [68]). These structures include (but are not limited to) the prefrontal cortex, limbic structures (e.g., amygdala, cingulate gyrus, orbitofrontal cortex), as well as white matter tracts connecting the cortical and subcortical regions. Damage to these structures can result in impairments in social behavior, recognition of emotions, empathy, judgment, decision-making. Consistent with these results, in our current study, we found correlations between social cognition measures (e.g., VINESOC) and AD in the PCR (which is a continuation of the fiber tracts that pass through the posterior limb of the internal capsule), as well as in fronto-parietal/occipital connections (SLF, IFO). Notably, lower FA values in the posterior limb of the internal capsule had been previously associated with schizotypy in VCFS [26] (and increased schizotypy implies more social difficulties).

### Limitations

While the atlas-based whole brain white matter analysis method is extremely valuable in automatically delineating ROIs, and it has been shown to have comparable reliability to manual tracing of ROIs, there are some limitations when using this method. More specifically, its accuracy could be decreased in the presence of certain brain abnormalities. For example, individuals with VCFS often have *enlarged ventricles*, and we observed that the AIR and LDDMM transformations did not always sufficiently warp the brain maps (especially for participants with very large ventricles) to match the JHU template, and thus, the corpus callosum ROIs sometimes included a portion of the lateral ventricles (esp. the splenum of the corpus callosum). This artifact could result in relatively noisy measurements of the corpus callosum ROIs, and, thus, loss of power. Indeed, in the current study, we did not find significant differences for the corpus callosum ROIs between individuals with VCFS and siblings. Another brain abnormality found more frequently in VCFS is *cavum septum pellucidum/vergae*, and 4 participants with VCFS in our current sample have this finding. As mentioned in the methods, a *cavum septum pellucidum* can significantly alter the anatomical location of the fornix. In order to avoid possible noise in the automated fornix delineation (in ABA), we have excluded the individuals with *cavum septum pellucidum/vergae* from our analyses of the fornix.

A relative strength (as well as potential weakness) of our study is that we correlated a large number of white matter tracts with neuropsychological measures. Therefore, we had to perform *corrections for multiple comparisons* in order to avoid Type 1 error. Yet, by lowering the p-value level for significance (by using FDR-correction), we might have missed some true correlations that would have been otherwise significant (if they were reported on their own/separately). While our current study is the largest DTI study individuals with VCFS so far, larger samples could result in higher statistical power and allow for the examination of the effects of the presence of psychiatric disorders or the use of medication on the DTI measures.

### Conclusions and Future Directions

Our results suggest abnormalities in the structural connectivity in a widespread cerebro-anatomical network, involving WM tracts in all cerebral lobes as well as the cerebellum (mostly evidenced by alterations in AD) in individuals with VCFS (relative to unaffected siblings), and correlations of WM microstructural measures to working memory, executive function, social cognition impairments, and/or IQ. These correlations may account for some of the major phenotypic features reported in VCFS. Future studies could focus on tractography of specific white matter tracts, genetic correlates (e.g., individual candidate genes in the 22q11.2 region) of WM alterations in VCFS, and the characterization of white matter abnormalities in individuals with VCFS and specific psychiatric diagnoses, including autism spectrum disorder (ASD). Last, longitudinal studies of individuals with VCFS in our current sample could explore whether the current DTI results (for individual participants) may have predictive power, as to who might later on develop schizophrenia/schizoaffective disorder.

## Endnotes

^a^A subset of the participants in this report has been included in an abstract for the 2011 Meeting of the Organization for Human Brain Mapping [69] and a presentation at the 2011 International Congress of Schizophrenia Research. All of the current participants have been included in an abstract presented at the 2011 Society for Neuroscience Meeting [70].

## Appendix

**Appendix 1** Table with original P-values (Orig P, not corrected for multiple comparisons), and FDR-corrected p-values (FDR P) from the MANOVAs on FA, AD and RD (in VCFS individuals vs. controls):

**Table:** 

**Tract**	**AD Orig P**	**AD FDR P**	**RD Orig P**	**RD FDR P**	**FA Orig P**	**FA FDR P**
**ACR_L**	***0.012***	***0.039***	N.S.	N.S.	N.S.	N.S.
**CGC_L**	***0.011***	***0.037***	N.S.	N.S.	N.S.	N.S.
**CGC_R**	***0.001***	***0.003***	***0.018***	N.S.	N.S.	N.S.
**CP_L**	N.S.	N.S.	N.S.	N.S.	***0.014***	N.S.
**EC_L**	***3.4E-04***	***0.002***	N.S.	N.S.	***0.022***	N.S.
**Fx_R**	***0.021***	N.S.	***0.019***	N.S.	***0.048***	N.S.
**Fx_L**	N.S.	N.S.	***0.037***	N.S.	N.S.	N.S.
**Fx/ST_L**	N.S.	N.S.	N.S.	N.S.	***0.013***	N.S.
**IFO_L**	***2.0E-04***	***0.001***	N.S.	N.S.	N.S.	N.S.
**IFO_R**	***5.6E-06***	***<0.001***	N.S.	N.S.	***0.029***	N.S.
**PCR_L**	***1.8E-08***	***<0.001***	***0.004***	N.S.	N.S.	N.S.
**PCR_R**	***9.2E-07***	***<0.001***	***0.001***	***0.038***	N.S.	N.S.
**PLIC_R**	N.S.	N.S.	***0.048***	N.S.	N.S.	N.S.
**PTR_L**	***3.0E-08***	***<0.001***	***0.018***	N.S.	N.S.	N.S.
**PTR_R**	***0.001***	***0.005***	***0.013***	N.S.	N.S.	N.S.
**RLIC_L**	***7.2E-05***	***0.001***	N.S.	N.S.	N.S.	N.S.
**RLIC_R**	N.S.	N.S.	***0.024***	N.S.	N.S.	N.S.
**SCP_L**	***3.5E-04***	***0.002***	N.S.	N.S.	N.S.	N.S.
**SCP_R**	***3.4E-04***	***0.002***	N.S.	N.S.	***0.045***	N.S.
**SCR_L**	***0.003***	***0.009***	***0.048***	N.S.	N.S.	N.S.
**SCR_R**	***0.015***	***0.046***	***0.003***	N.S.	N.S.	N.S.
**SFO_L**	N.S.	N.S.	N.S.	N.S.	***0.025***	N.S.
**SLF_L**	***3.3E-06***	***<0.001***	***0.020***	N.S.	N.S.	N.S.
**SLF_R**	***5.2E-09***	***<0.001***	***0.024***	N.S.	N.S.	N.S.
**SS_L**	***2.6E-04***	***0.002***	N.S.	N.S.	N.S.	N.S.
**SS_R**	***0.038***	N.S.	N.S.	N.S.	N.S.	N.S.
**UNC_L**	N.S.	N.S.	N.S.	N.S.	***0.001***	***0.020***
**UNC_R**	N.S.	N.S.	***0.036***	N.S.	***0.001***	***0.020***

## Abbreviations

CST: Corticospinal tract; ICP: Inferior cerebellar peduncle; ML: Medial lemniscus; SCP: Superior cerebellar peduncle; CP: Cerebral peduncle; ALIC: Anterior limb of the internal capsule; PLIC: Posterior limb of the internal capsule; PTR: Posterior thalamic radiation (include optic radiation); ACR: Anterior corona radiata; SCR: Superior corona radiata; PCR: Posterior corona radiata; CGC: Cingulum (cingulate gyrus); CGH: Cingulum (hippocampus); Fx/ST: Fornix (cres)/Stria terminalis (can not be resolved with current resolution); SLF: Superior longitudinal fasciculus; SFO: Superior fronto-occipital fasciculus (could be a part of anterior internal capsule); IFO: Inferior fronto-occipital fasciculus; SS: Sagittal stratum (include inferior longitudinal fasciculus and inferior fronto-occipital fasciculus); EC: External capsule; UNC: Uncinate fasciculus; PCT: Pontine crossing tract (a part of MCP); MCP: Middle cerebellar peduncle; Fx: Fornix (column and body of the fornix); GCC: Genu of the corpus callosum; BCC: Body of the corpus callosum; SCC: Splenium of the corpus callosum; RLIC: Retrolenticular part of the internal capsule; ABA: Atlas-based analysis; AD: Axial diffusivity; A/P Long Tracts: Anterior-Poster Long Tracts; ASD: Autism spectrum disorder; BASC-2: Behavior Assessment System for Children 2^nd^ ed; BRIEF: Behavior Rating Inventory of Executive Functioning; CGAS: Children's Global Assessment Scale; CVLT: California verbal learning test; DTI: Diffusion tensor imaging; FA: Fractional anisotropy; RD: Radial diffusivity; SRS: Social Responsiveness Scale; VBM: Voxel-based morphometry; VCFS: Velo-cardio-facial syndrome; WCST: Wisconsin card sorting test; WM: White matter.

## Competing interests

The authors declare that they have no competing interests.

## Authors’ contributions

PR participated in data analysis and wrote the first draft of the manuscript. KA and WF conducted the neuropsychological and psychiatric assessments of the participants, and KA contributed to the draft of the manuscript. IC participated in the design of the study and in data analysis, and contributed to the draft of the manuscript. CS and AK participated in data analysis. DW assisted with statistical analysis. RS participated in the overall conceptualization of the study, and contributed to the draft of the manuscript. WK conceived of the study design, participated in data analysis, and helped to draft the manuscript. All authors read and approved the final manuscript.
